# Supplementary material for: Environmental enrichment and physical exercise prevent stress-induced social avoidance and blood-brain barrier alterations via Fgf2
Source: Nat Commun. 2026 Jan 16;17:1297. doi: 10.1038/s41467-025-68058-9 (PMC12868728; doi:10.1038/s41467-025-68058-9)
Supplement: Supplementary file 1 — Supplementary Information [file 41467_2025_68058_MOESM1_ESM.pdf]

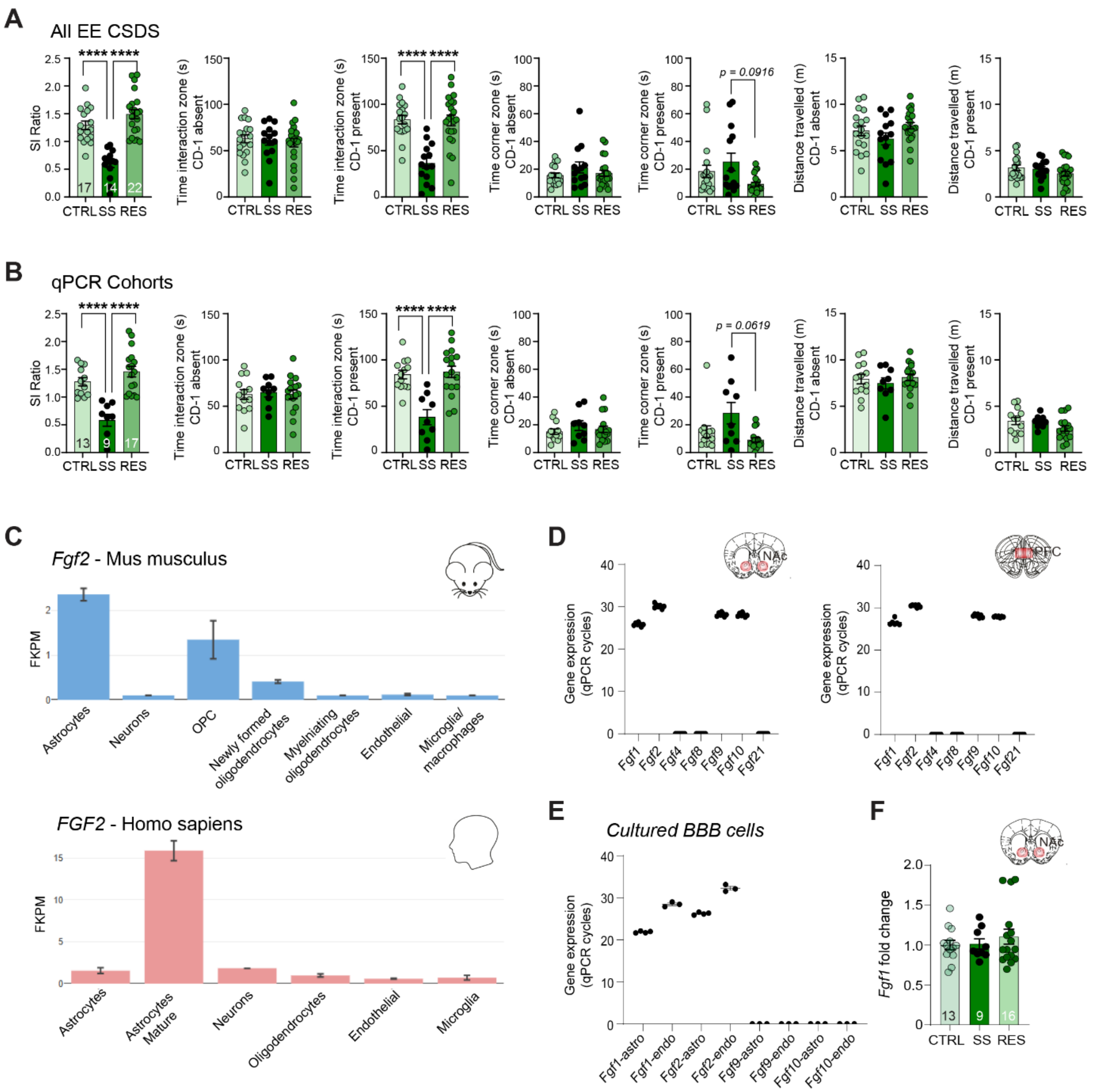

(Adapted from https://brainrnaseq.org/ of Zhang et al., 2014)

Supplementary Figure 1. Additional behavioral and transcriptional data for male mice with access to an enriched environment. Additional behavioral metrics are shown for social interaction (SI) tests of male mice with enriched environment (EE) after CSDS for all cohorts grouped (**A**, ****$p<0.0001$ for SI ratio and time spent in the interaction zone when the CD-1 is present), and then split by tissue use, first for qPCR (**B**, ****$p<0.0001$ for SI ratio and time spent in the interaction zone when the CD-1 is present). **C**, Cell-specific *Fgf2* gene expression in mice and human according to atlases. **D**, Gene expression of various *Fgf*s in the male nucleus accumbens (NAc) and prefrontal cortex (PFC). **E**, Gene expression of brain most abundant *Fgf*s in cultured astrocytes or endothelial cells. **F**, Exposure to 10-day chronic social defeat stress does not affect *Fgf*1 expression in the NAc of male mice. Data represent mean ± s.e.m., the number of animals is indicated on graphs. Group comparisons were evaluated with one- or two-way ANOVA followed by Bonferroni's posttests; *$p<0.05$, **$p<0.01$, ***$p<0.001$, ****$p<0.0001$.

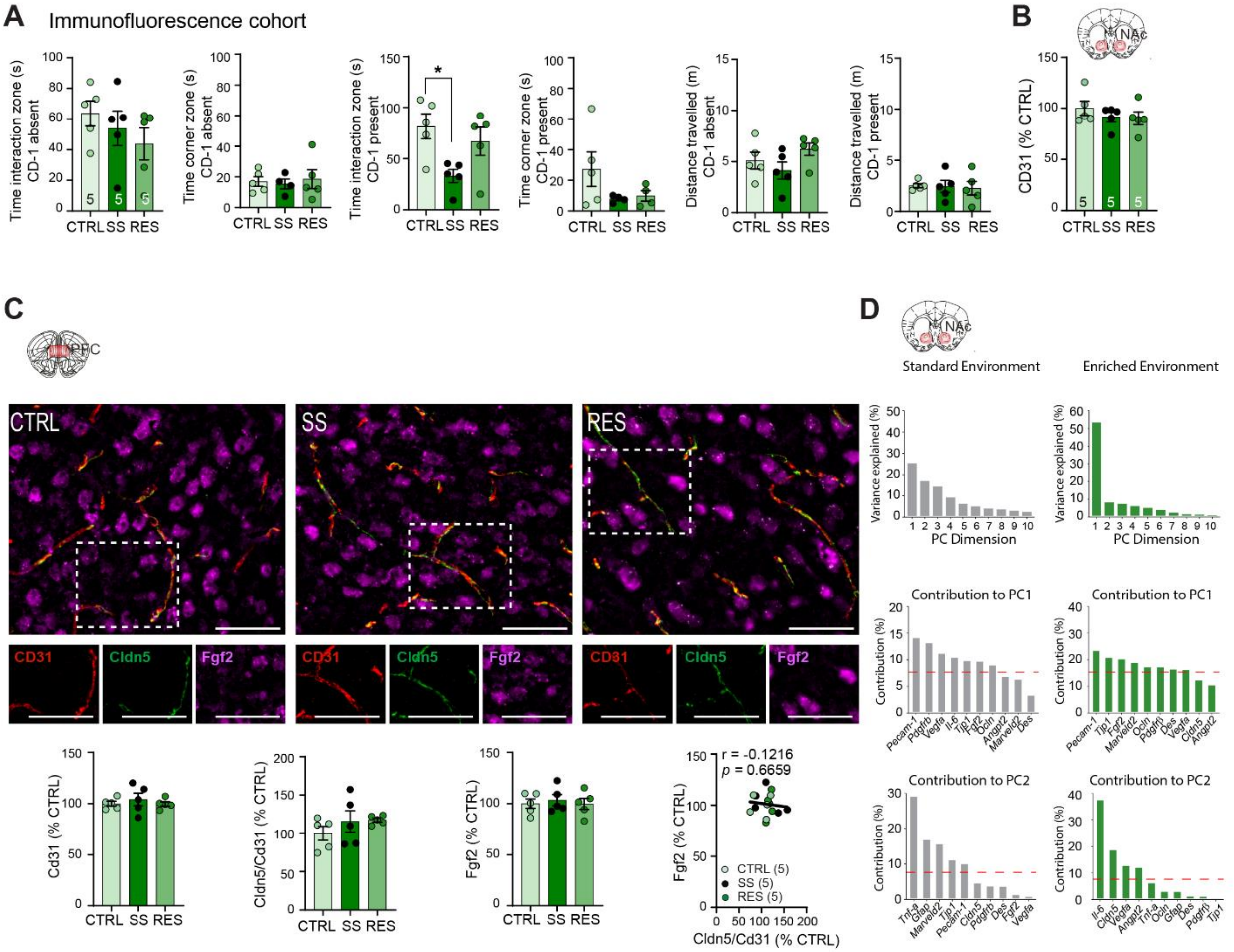

Supplementary Figure 2. Additional behavioral, morphological, and statistical data for male mice with access to an enriched environment. A, Additional behavioral metrics are shown for social interactions of male mice with access to an enriched environment after CSDS for the cohort used for immunofluorescence. **B,** Staining for CD31, a blood vessel marker, in the male NAc is not affected by CSDS with EE access. **C**, No substantial changes are observed in immunofluorescent labelling of Cd31, Cldn5, or Fgf2 in male PFC after 10 d CSDS with EE (scalebar = 50 µm). **D**, Contribution of principal component (PC) dimensions and genes involved in PC1 and PC2 as determined by principal component analysis (PCA) of qPCR datasets from male NAc following 10 d CSDS in standard environment (Menard et al., 2017) and EE. Data represent mean ± s.e.m., the number of animals is indicated on graphs. Group comparisons were evaluated with one- or two-way ANOVA followed by Bonferroni's posttests; *p<0.05, **p<0.01, ***p<0.001, ****p<0.0001.

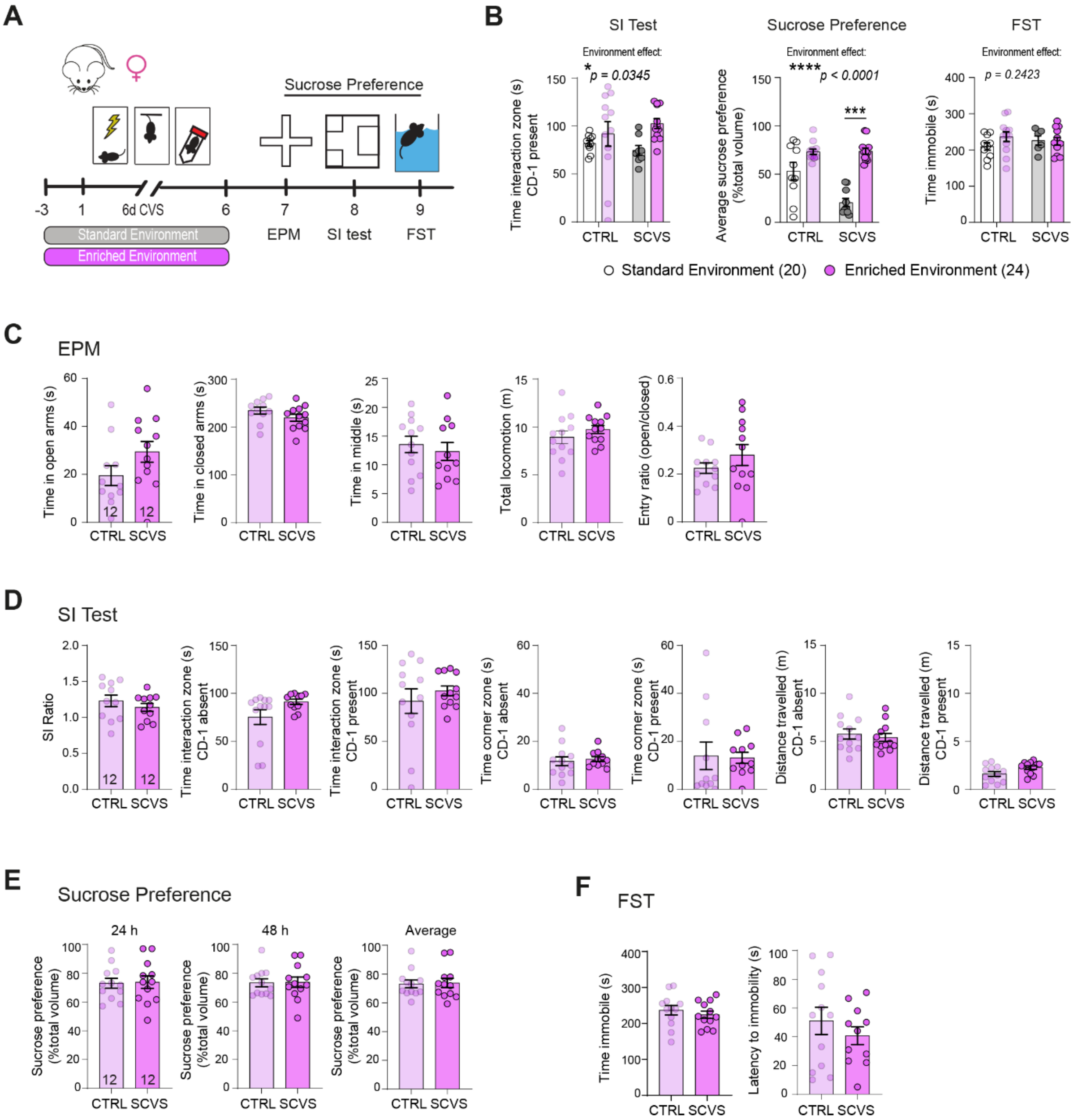

Supplementary Figure 3. Additional behavioral data for female mice with access to an enriched environment. **A,** Experimental timeline for assessing depressive and anxiety-like behavior after subchronic variable stress (SCVS) with enriched environment (EE). Female mice were housed with a nestlet, plastic chew toy, and shelter beginning 3 d prior to stress and continuing until the last session, followed by testing with elevated plus maze (EPM), social interaction (SI) tests, sucrose preference, and the forced swim test (FST). **B,** Compared to previously published findings (Dion-Albert, 2022), EE ameliorates SCVS-induced deficits in the SI test and sucrose preference. Additional behavioral metrics are presented for the EPM (**C**), SI test (**D**), sucrose preference test (**E**), and FST (**F**). Data represent mean ± s.e.m., the number of animals is indicated on graphs. Group comparisons were evaluated with one- or two-way ANOVA followed by Bonferroni's posttests, or two-tailed t-test with Welch's correction where appropriate; *p<0.05, **p<0.01, ***p<0.001, ****p<0.0001.

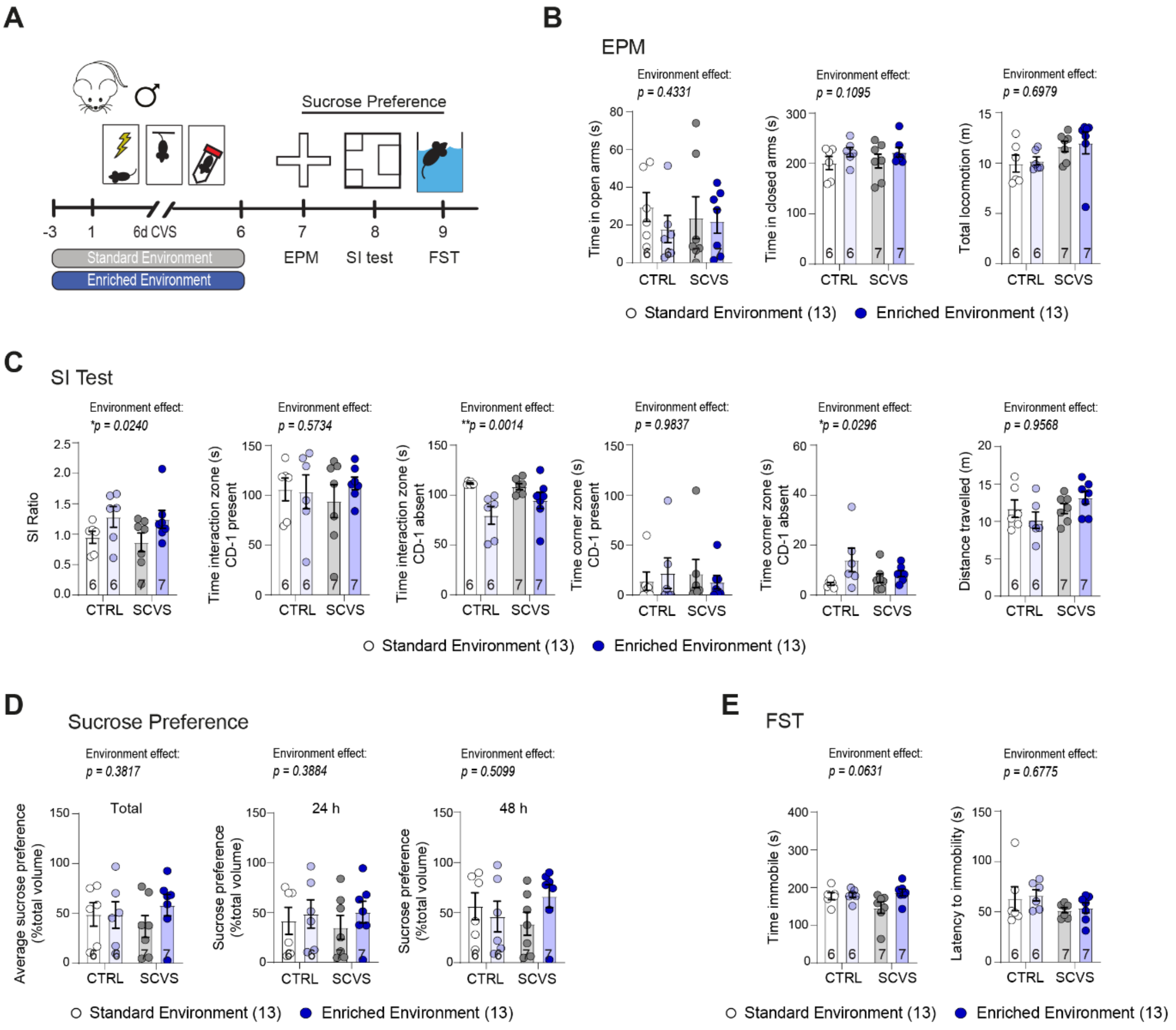

Supplementary Figure 4. Behavioral data for male mice with access to an enriched environment and exposed to 6-d SCVS. A, Experimental timeline for assessing depressive and anxiety-like behavior after subchronic variable stress (SCVS) with enriched environment (EE). Male mice were housed with a nestlet, plastic chew toy, and shelter beginning 3 d prior to stress and continuing until the last session, followed by testing with elevated plus maze (EPM), social interaction (SI) tests, sucrose preference, and the forced swim test (FST). Behavioral metrics are presented for the EPM (**B**), SI test (**C**), sucrose preference test (**D**), and FST (**E**). Data represent mean ± s.e.m., the number of animals is indicated on graphs. Group comparisons were evaluated with one- or two-way ANOVA followed by Bonferroni's posttests, or two-tailed t-test with Welch's correction where appropriate; *p<0.05.

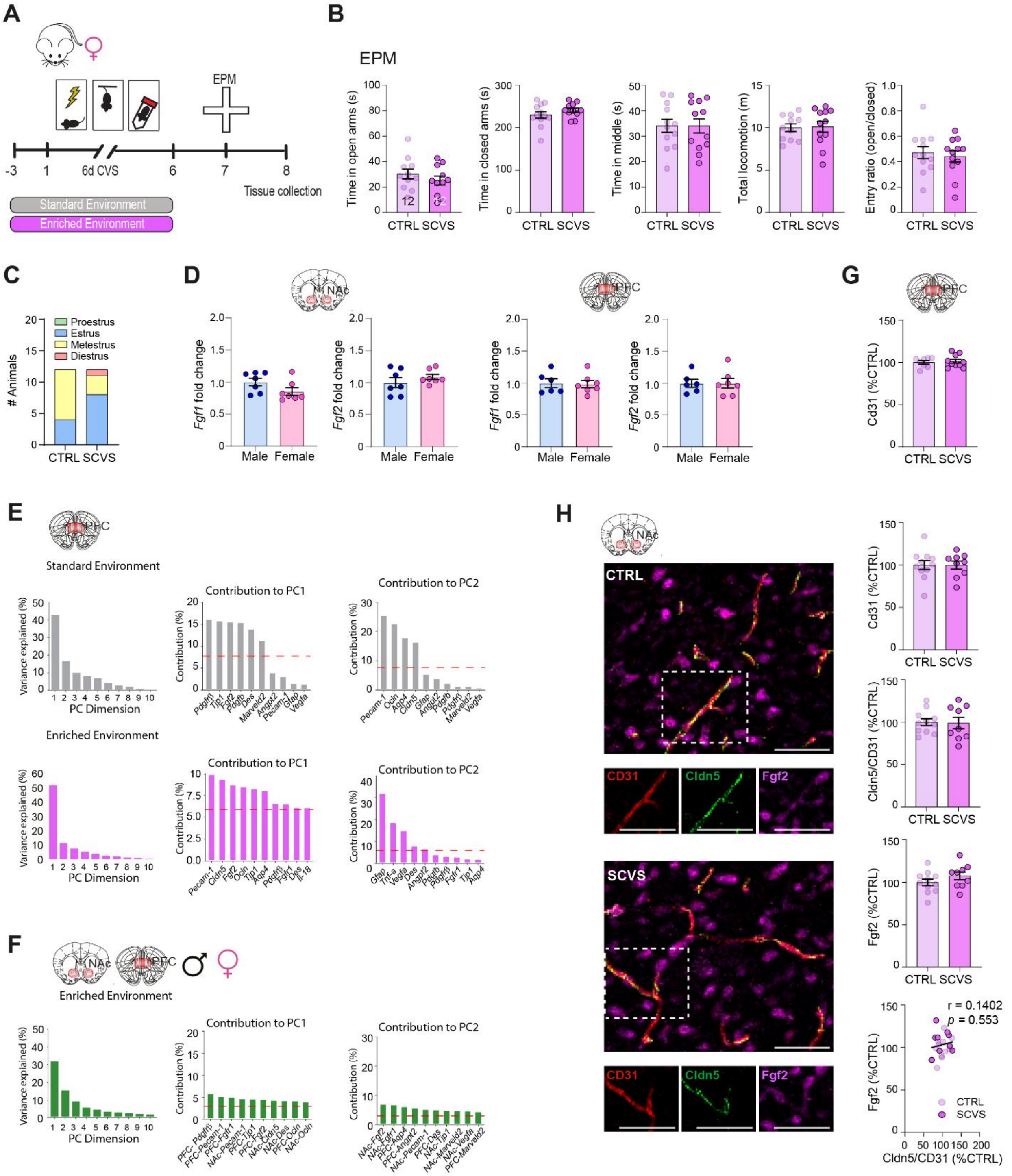

Supplementary Figure 5. Additional behavioral, morphological, and statistical data for female mice with access to an enriched environment. A, Experimental timeline for SCVS with access to enriched environment (EE), followed by EPM testing and tissue collection. Female mice were housed with a nestlet, plastic chew toy, and shelter beginning 3 d prior to stress and continuing until the last session. **B**, Additional behavioral metrics from EPM testing of this cohort. **C**, Estrus cycle stage determined at sacrifice in CTRL and SCVS groups. **D**, Expression of *Fgf1* and *Fgf2* in the nucleus accumbens (NAc) and prefrontal cortex (PFC) of naïve male and female mice. **E**, Contribution of principal component (PC) dimensions and genes involved in PC1 and PC2 as determined by principal component analysis

(PCA) of qPCR datasets from female NAc following SCVS in standard environment (Dion-Albert et al., 2022) and EE. **F,** Contribution of principal component (PC) dimensions and genes involved in PC1 and PC2 as determined by principal component analysis (PCA) of qPCR datasets from both NAc + PFC of males and females following SCVS with access to EE. **G,** No change in Cd31 immunolabelling in the female PFC post-SCVS. **H,** No significant changes in Cd31, Cldn5, or Fgf2 immunolabelling in the female NAc following SCVS with EE (scalebar = 50 μm). Data represent mean ± s.e.m., the number of animals is indicated on graphs. Group comparisons were evaluated with two-tailed t-test with Welch's correction where appropriate.

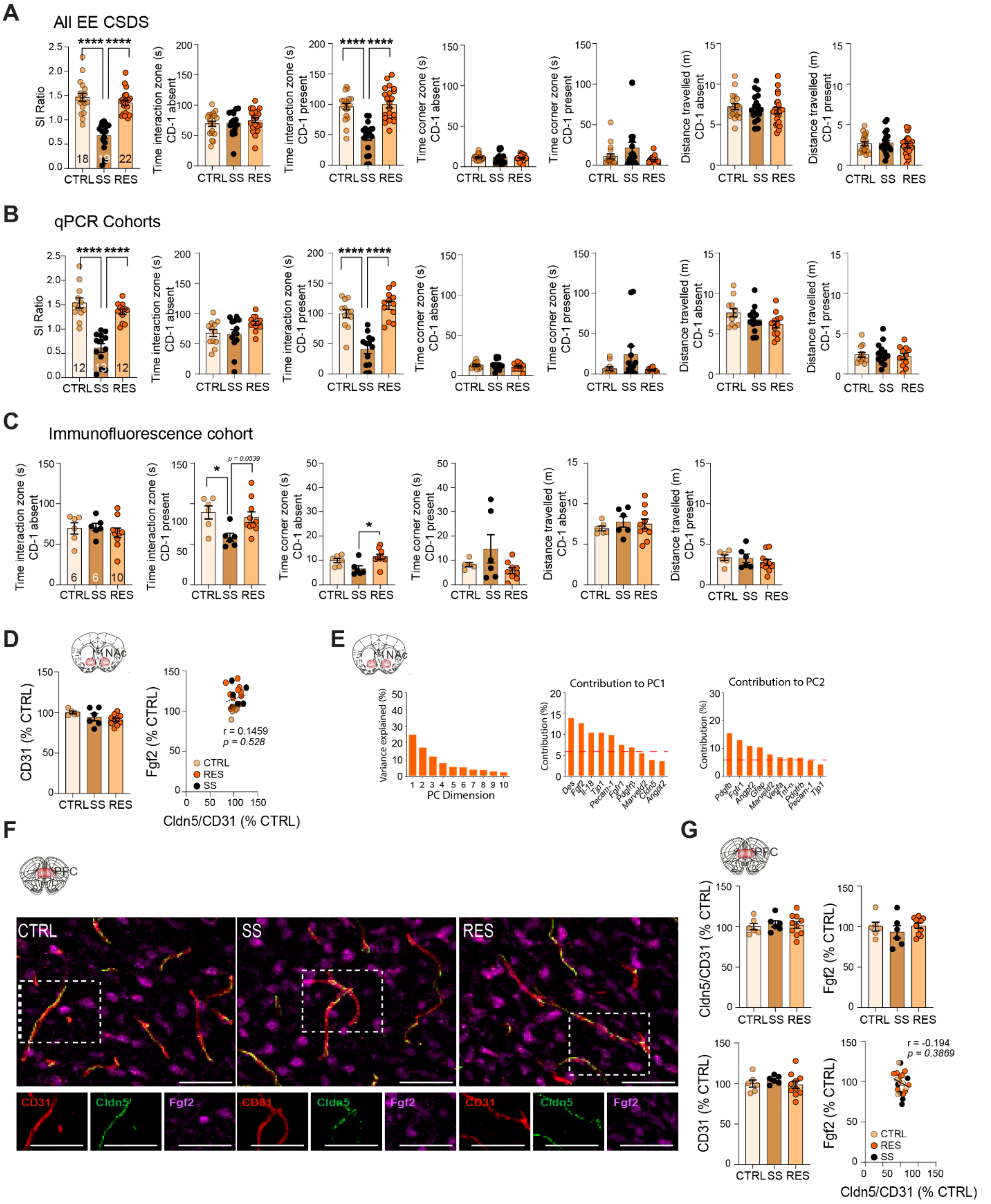

Supplementary Figure 6. Additional behavioral, morphological, and statistical data for male mice with access to physical exercise. Additional behavioral metrics are shown from social interaction (SI) tests of male mice with physical exercise (PE) after CSDS for all cohorts grouped (**A**, ****p<0.0001 for SI ratio and time spent in the interaction zone when the CD-1 is present), and then split by tissue use, qPCR (**B**, ****p<0.0001 for SI ratio and time spent in the interaction zone when the CD-1 is present) and immunofluorescence (**C**, *p=0.0207 for time spent in the

interaction zone when the CD-1 is present and **p=0.0016 for time spent in the corners when the CD-1 is absent). **D,** Staining for CD31, a blood vessel marker, in the male NAc is not affected by CSDS with EE access, and Cldn5 levels do not correlate with Fgf2. **E**, Contribution of principal component (PC) dimensions and genes involved in PC1 and PC2 as determined by principal component analysis (PCA) of qPCR datasets from male NAc following 10 d CSDS in standard environment (Menard et al., 2017) and EE. **F**, Representative immunofluorescent images of Cd31, Cldn5, and Fgf2 in male PFC after 10 d CSDS (scalebar = 50 µm) and **G,** No substantial changes are observed in immunofluorescent labelling of these markers. Data represent mean ± s.e.m., the number of animals is indicated on graphs. Group comparisons were evaluated with one- or two-way ANOVA followed by Bonferroni's posttests; *p<0.05, **p<0.01, ***p<0.001, ****p<0.0001.

A

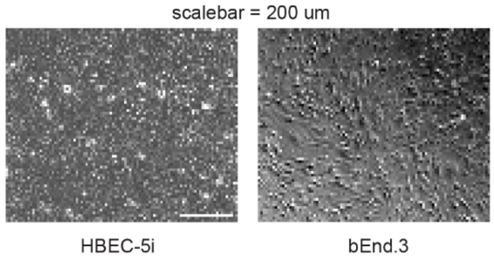

B

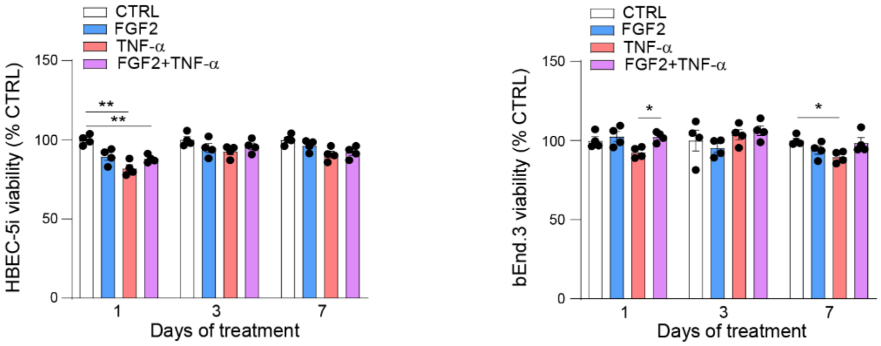

C

HBEC-5i (human)		Baseline		Day 1		Day 3		Day 7	
		Mean	St. Dev	Mean	St. Dev	Mean	St. Dev	Mean	St. Dev
TEER (Ω*cm^2)	CTRL (8)	36.825	8.32582	37.725	7.435	42.975	12.6262	41.625	12.2164
	FGF2 (8)	38.175	7.64979	38.25	6.40424	41.625	10.8945	43.425	11.0678
	TNF-α (8)	39.225	8.24513	37.65	5.73486	38.325	10.0779	39.45	12.2618
	FGF2+TNF-α (8)	39.225	8.81196	42	5.22084	43.725	10.6245	45.525	15.2783

bEnd.3 (mouse)		Baseline		Day 1		Day 3		Day 7	
		Mean	St. Dev	Mean	St. Dev	Mean	St. Dev	Mean	St. Dev
TEER (Ω*cm^2)	CTRL (8)	21.6	3.36367	22.875	4.11261	27	2.29035	28.35	1.99643
	FGF2 (8)	20.25	2.62025	22.425	4.74545	28.125	3.5632	29.475	3.62048
	TNF-α (8)	22.725	1.71026	21.6	3.57131	25.8	2.44949	27.975	2.37712
	FGF2+TNF-α (8)	22.875	2.74838	23.7	5.14143	28.425	4.7346	30.375	3.15764

Supplementary Figure 7. Additional morphological and cell viability data for HBEC human and bEnd.3 mouse endothelial cells. A, Representative brightfield images of HBEC-5i and bEnd.3 cells demonstrating endothelial morphology, scalebar = 200 µm. **B,** HBEC-5i and bEnd.3 cell viability is not substantially altered by 7 d treatment with FGF2 and/or TNF-α with a significant treatment effect observed only at day 1 for HBEC cells (**p=0.0032). **C,** Raw TEER measurements for HBEC-5i and bEnd.3.

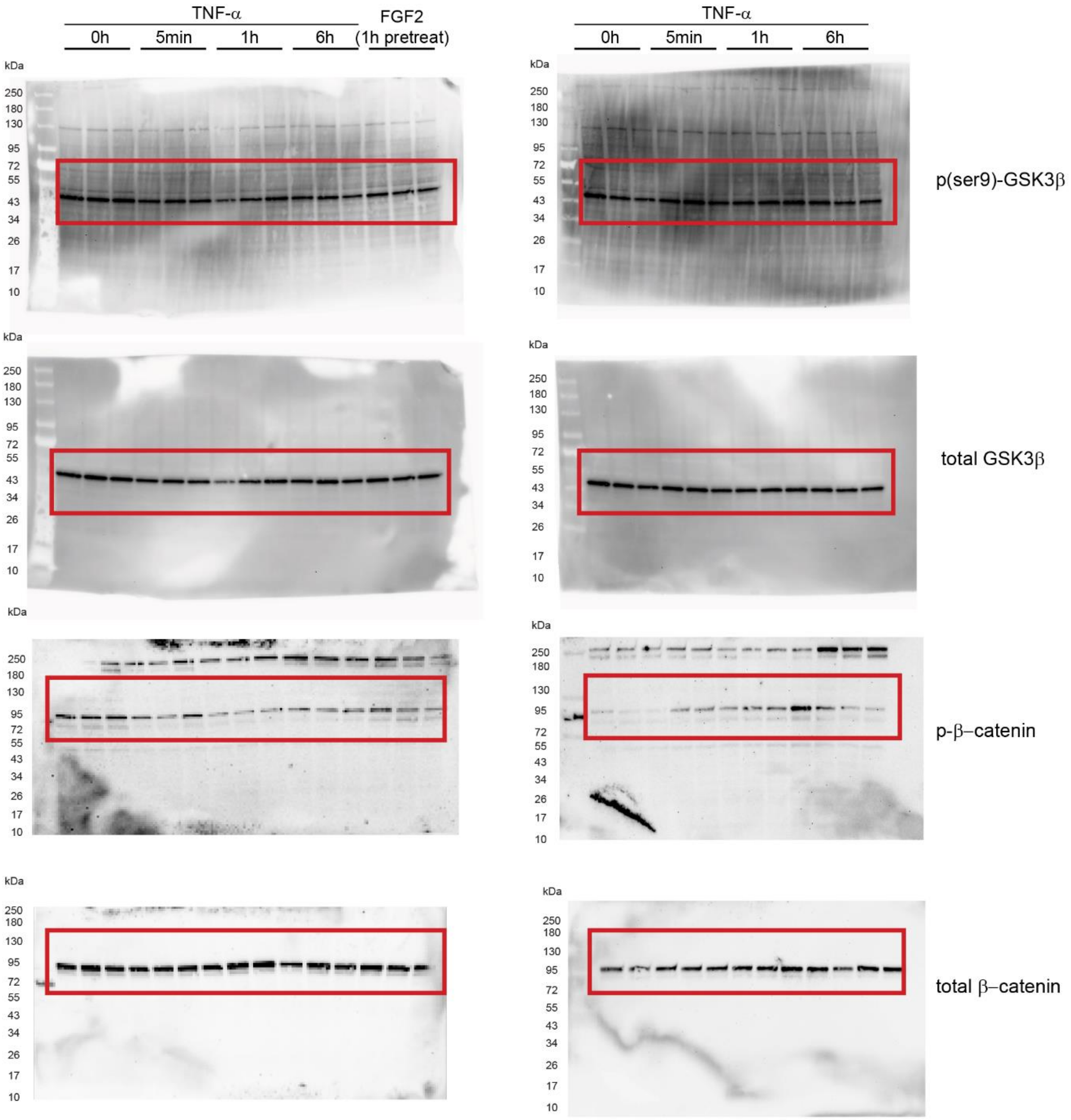

Supplementary Figure 8. Full-length Western Blots. Full-length Western blots of HBEC-5i cells treated with tumor necrosis factor alpha (TNF-α) or fibroblast growth factor 2 (FGF2). Protein levels were evaluated for glycogen synthase kinase-3 beta (GSK3β) serine 9 residue (p-ser9)-GSKβ, total GSK3β, phospho-beta-catenin (p-β-catenin) and finally, total β-catenin.

Supplementary Table 1. Primers for RT-qPCR.

Gene	Species	Ref Seq #	Assay ID	Forward Primer (5'-3')	Reverse Primer (5'-3')
Gapdh	Mouse	NM_008084(1)	Mm.PT.39a.1	PrimeTime® qPCR Primers Exon Location 2 - 3	
Vegfa	Mouse	NM_001025250(3)	Mm.PT.58.14200306	PrimeTime® qPCR Primers Exon Location 1 - 2	
Fgf2	Mouse	NM_008006(1)	Mm.PT.56a.5129235	PrimeTime® qPCR Primers Exon Location 1 - 3	
Pdgfb	Mouse	NM_011057(1)	Mm.PT.58.32585335	PrimeTime® qPCR Primers Exon Location 2 - 3	
Pecam-1	Mouse	NM_001032378(2)	Mm.PT.58.43167370	PrimeTime® qPCR Primers Exon Location 7 – 8	
Fgfr1	Mouse	NM_001079908(3)	Mm.PT.58.6948463	PrimeTime® qPCR Primers Exon Location 6 - 7	
Angpt2	Mouse	NM_007426(1)	Mm.PT.58.29139310	PrimeTime® qPCR Primers Exon Location 1a - 2	
Cldn5	Mouse	NM_013805(1)		TTTCTTCTATGCGCAGTTGG	GCAGTTTGGTGCCTACTTCA
Ocln	Mouse	NM_008756(1)	Mm.PT.58.42749240	PrimeTime® qPCR Primers Exon Location 7 - 9	
Tjp1	Mouse	NM_001163574(2)	Mm.PT.58.29459730	PrimeTime® qPCR Primers Exon Location 18 - 19	
Marveld2	Mouse	NM_001038602(2)	Mm.PT.58.7719303	PrimeTime® qPCR Primers Exon Location 5 - 7	
Gfap	Mouse	NM_010277(1)	Mm.PT.58.31297710	PrimeTime® qPCR Primers Exon Location 6 - 9	
Aqp4	Mouse	NM_009700(1)	Mm.PT.58.9080805	PrimeTime® qPCR Primers Exon Location 1 - 2	
Pdgfrb	Mouse	NM_001146268(2)	Mm.PT.56a.5869521	PrimeTime® qPCR Primers Exon Location 23 - 24	
Des	Mouse	NM_010043(1)	Mm.PT.58.13181631	PrimeTime® qPCR Primers Exon Location 7 - 9	
Il-6	Mouse	NM_031168.2		TAGTCCTTCCTACCCCAATTTCC	TTGGTCCTTAGCCACTCCTTC
Il-18	Mouse	NM_008360.2		GACTCTTGCGTCAACTTCAAGG	CAGGCTGTCTTTTGTCAACGA
Tnf-α	Mouse	NM_013693.3		CCCTCACACTCAGATCATCTTCT	GCTACGACGTGGGCTACAG
Vcam1	Mouse	NM_011693(1)	Mm.PT.58.9687546	PrimeTime® qPCR Primers Exon Location 5 - 6	
Ripk1	Mouse	NM_009068(1)	Mm.PT.58.7201430	PrimeTime® qPCR Primers Exon Location 4 - 5	
Hdac1	Mouse	NM_008228(1)	Mm.PT.58.14183463	PrimeTime® qPCR Primers Exon Location 7 - 8	
Foxo1	Mouse	NM_019739(1)	Mm.PT.58.6477586	PrimeTime® qPCR Primers Exon Location 1 - 2	
Gsk3β	Mouse	NM_019827(1)	Mm.PT.58.41280327	PrimeTime® qPCR Primers Exon Location 6 - 7	
Fgf1	Mouse	NM_010197(1)	Mm.PT.56a.41158563	PrimeTime® qPCR Primers Exon Location 1-2	
Fgf4	Mouse	NM_010202(1)	Mm.PT.58.7358007.gs	PrimeTime® qPCR Primers Exon Location 2-3	
Fgf8	Mouse	NM_001166361	Mm.PT.58.33374299	PrimeTime® qPCR Primers Exon Location 4-5	
Fgf9	Mouse	NM_013518(1)	Mm.PT.56a.5456225	PrimeTime® qPCR Primers Exon Location 1-2	
Fgf10	Mouse	NM_008002(1)	Mm.PT.58.11905869	PrimeTime® qPCR Primers Exon Location 1-2	
Fgf21	Mouse	NM_020013(1)	Mm.PT.58.29365871.g	PrimeTime® qPCR Primers Exon Location 1-1	
GAPDH	Human	NM_002046(1)	Hs.PT.39a.22214836	PrimeTime® qPCR Primers Exon Location 2 - 3	
CLDN5	Human	NM_001130861(2)	Hs.PT.58.1483777.g	PrimeTime® qPCR Primers Exon Location 1 - 1[1]	
OCLN	Human	NM_001205254(3)	Hs.PT.58.15235048	PrimeTime® qPCR Primers Exon Location 6 - 7	
RIPK1	Human	NM_003804(1)	Hs.PT.58.15545621	PrimeTime® qPCR Primers Exon Location 7 - 8	
HDAC1	Human	NM_004964(1)	Hs.PT.58.38680914	PrimeTime® qPCR Primers Exon Location 3 - 4	
FOXO1	Human	NM_002015(1)	Hs.PT.58.40005627	PrimeTime® qPCR Primers Exon Location 1 - 2	
GSK3β	Human	NM_001146156(2)	Hs.PT.58.40111551	PrimeTime® qPCR Primers Exon Location 6 - 7	
FGFR1	Human	NM_001174067(1)	Hs.PT.58.15035470	PrimeTime® qPCR Primers Exon Location 3 - 4	
FGF2	Human	NM_002006(1)	Hs.PT.58.24613308	PrimeTime® qPCR Primers Exon Location 1 - 2	
VCAM-1	Human	NM_001199834(3)	Hs.PT.58.20405152	PrimeTime® qPCR Primers Exon Location 2a - 3	
IL-1β	Human	NM_000576(1)	Hs.PT.58.1518186	PrimeTime® qPCR Primers Exon Location 1 - 3	
IL-6	Human	NM_000600(1)	Hs.PT.58.40226675	PrimeTime® qPCR Primers Exon Location 4 - 5	

Supplementary Table 2. Primary and secondary antibodies

Immunofluorescence				
**Target**	**Company**	**Cat #**	**Host**	**Dilution**
Cd31	Invitrogen	14-0311-85	Rat	1:100
TL DyLight 488	Vector Laboratories	DL-1174-1	-	1:100
Cldn5	Invitrogen	34-1600	Rabbit	1:250
Fgf2	Biosensis	10782-612	Sheep	1:200
Fgf2	Abcam	ab208687	Rabbit	1:250
Gfap	Cedarlane (Synaptic system)	173004/173308	Guinea pig	1:300
Aldh1l1	Novus	NBP250033	Mouse	1:700
Cy2 Anti-Rat	Jackson ImmunoResearch	712-175-153	Donkey	1:400
Cy3 Anti-Rabbit	Jackson ImmunoResearch	711-225-152	Donkey	1:400
Cy3 Anti-Rat	Jackson ImmunoResearch	712-165-153	Donkey	1:400
Cy3 Anti-Sheep	Jackson ImmunoResearch	713-165-147	Donkey	1:400
Cy5 Anti-Sheep	Jackson ImmunoResearch	713-175-147	Donkey	1:400
Cy5 Anti-Rabbit	Jackson ImmunoResearch	711-175-152	Donkey	1:400
Cy5 Anti-Guinea Pig	Jackson ImmunoResearch	706-175-148	Donkey	1:400
Alexa Fluor 647 Anti-mouse	Jackson ImmunoResearch	715-605-151	Donkey	1:400
**Western Blot**				
**Target**	**Company**	**Cat #**	**Host**	**Dilution**
p(ser9)-Gsk3β	Cell Signalling	9336	Rabbit	1:1000
Gsk3β	Cell Signalling	9315	Rabbit	1:1000
p-β-Catenin	Cell Signalling	9562	Rabbit	1:1000
β-Catenin	Cell Signalling	9561	Rabbit	1:1000
Cldn5	Invitrogen	34-1600	Rabbit	1:1000
Anti-Rabbit IgG, HRP	Cell Signalling	7074	Goat	1:5000

Supplementary Table 3. Demographic and sociodemographic data of the human cohort.

Sex	Dx	Severity	University diploma	Employed	2 > Languages
Women	Control	Mild	No	No	Yes
Women	Control	Minimal	Yes	No	Yes
Women	Control	Minimal	No	Yes	No
Women	Control	Minimal	No	No	No
Women	Control	Minimal	No	Yes	Yes
Women	Control	Minimal	No	Yes	Yes
Women	Control	Minimal	No	No	No
Women	Control	Minimal	No	No	No
Men	Control	Minimal	No	No	No
Women	Control	Minimal	No	No	Yes
Women	Control	Minimal	No	Yes	Yes
Men	Control	Minimal	No	Yes	Yes
Women	Control	Mild	No	No	No
Women	Control	Minimal	Yes	No	Yes
Women	Control	Minimal	No	No	Yes
Women	Control	Minimal	No	Yes	Yes
Women	Control	Minimal	Yes	No	Yes
Men	Control	Minimal	No	No	No
Men	Control	Mild	No	Yes	Yes
Women	Control	Minimal	No	Yes	Yes
Women	Control	Minimal	No	No	Yes
Men	Control	Minimal	No	Yes	Yes
Women	Control	Mild	Yes	Yes	No
Men	Control	Minimal	No	No	Yes
Men	Control	Minimal	No	No	No
Men	Control	Minimal	No	No	No
Women	Control	Minimal	Yes	No	Yes
Women	Control	Minimal	Yes	Yes	No
Men	Control	Minimal	No	No	Yes
Women	Control	Minimal	No	Yes	No
Women	Control	Minimal	No	Yes	No
Women	Control	Minimal	Yes	Yes	No
Men	Control	Mild	No	Yes	No
Men	Control	Minimal	No	Yes	Yes
Women	Control	Minimal	No	No	Yes
Men	Control	Minimal	No	No	Yes
Men	Control	Minimal	Yes	No	Yes
Men	Control	Minimal	Yes	No	Yes
Men	Control	Minimal	No	No	Yes
Men	Control	Minimal	No	No	Yes

Men	Control	Minimal	No	Yes	Yes
Men	Control	Minimal	No	Yes	Yes
Men	Control	Minimal	Yes	No	Yes
Men	Control	Minimal	No	No	Yes
Women	Control	Minimal	No	No	Yes
Men	Control	Minimal	No	No	No
Men	Control	Minimal	No	No	Yes
Women	Depression	Moderately severe	Yes	No	No
Men	Depression	Severe	Yes	No	Yes
Men	Depression	Moderately severe	No	No	Yes
Men	Depression	Moderate	No	No	No
Men	Depression	Severe	No	No	Yes
Men	Depression	Moderately severe	No	No	No
Men	Depression	Severe	No	No	Yes
Women	Depression	Moderately severe	No	No	Yes
Women	Depression	Moderately severe	No	No	No
Men	Depression	Moderate	No	No	No
Women	Depression	Severe	No	No	No
Men	Depression	Severe	No	No	Yes
Men	Depression	Severe	No	No	No
Men	Depression	Severe	Yes	No	Yes
Women	Depression	Moderately severe	No	No	No
Men	Depression	Moderate	No	No	No
Men	Depression	Severe	No	No	No
Men	Depression	Severe	No	No	Yes
Women	Depression	Moderate	No	Yes	Yes
Women	Depression	Moderately severe	No	No	Yes
Men	Depression	Severe	No	No	No
Men	Depression	Moderately severe	No	No	No
Women	Depression	Severe	No	Yes	Yes
Men	Depression	Severe	No	No	Yes
Men	Depression	Moderate	No	No	No
Women	Depression	Severe	No	No	Yes
Men	Depression	Severe	No	No	No
Women	Depression	Severe	No	Yes	Yes
Women	Depression	Severe	No	No	No
Men	Depression	Severe	No	No	No
Women	Depression	Severe	No	No	Yes
Men	Depression	Severe	No	No	Yes
Women	Depression	Severe	No	No	No
Men	Depression	Severe	No	No	No
Men	Depression	Severe	No	No	No
Men	Depression	Severe	Yes	No	No

Women	Depression	Severe	No	No	Yes
Women	Depression	Moderate	Yes	No	No
Women	Depression	Moderately severe	No	No	Yes
Men	Depression	Moderately severe	No	No	Yes
Men	Depression	Moderately severe	No	No	Yes
Men	Depression	Moderately severe	No	Yes	Yes
Women	Depression	Moderately severe	No	No	Yes
Women	Depression	Moderate	No	No	Yes
Women	Depression	Severe	No	No	No
Women	Depression	Moderately severe	No	Yes	No
Men	Depression	Severe	No	No	No
Women	Depression	Severe	No	Yes	Yes
Men	Depression	Severe	No	Yes	Yes
Women	Depression	Moderate	No	No	No
Women	Depression	Severe	No	No	No
Men	Depression	Moderately severe	No	Yes	Yes
Men	Depression	Severe	No	No	No
Women	Depression	Severe	No	No	Yes
Men	Depression	Moderately severe	Yes	No	Yes
Men	Depression	Severe	No	Yes	No
Women	Control	Mild	No	No	Yes
Women	Control	Minimal	Yes	No	Yes
Women	Control	Minimal	No	Yes	No
Women	Control	Minimal	No	No	No
Women	Control	Minimal	No	Yes	Yes
Women	Control	Minimal	No	Yes	Yes
Women	Control	Minimal	No	No	No
Women	Control	Minimal	No	No	No
Men	Control	Minimal	No	No	No
Women	Control	Minimal	No	No	Yes
