# Supplementary material for: Environmental enrichment and physical exercise prevent stress-induced social avoidance and blood-brain barrier alterations via Fgf2
Source: Nat Commun. 2026 Jan 16;17:1297. doi: 10.1038/s41467-025-68058-9 (PMC12868728; doi:10.1038/s41467-025-68058-9)
Supplement: Supplementary file 2 — Reporting Summary [file 41467_2025_68058_MOESM2_ESM.pdf]

nature portfolio

Corresponding author(s): Caroline Menard

Last updated by author(s): Sep 9, 2025

Reporting Summary

Nature Portfolio wishes to improve the reproducibility of the work that we publish. This form provides structure for consistency and transparency in reporting. For further information on Nature Portfolio policies, see our Editorial Policies and the Editorial Policy Checklist.

Statistics

For all statistical analyses, confirm that the following items are present in the figure legend, table legend, main text, or Methods section.

n/a	Confirmed	
☐	☒	The exact sample size (*n*) for each experimental group/condition, given as a discrete number and unit of measurement
☐	☒	A statement on whether measurements were taken from distinct samples or whether the same sample was measured repeatedly
☐	☒	The statistical test(s) used AND whether they are one- or two-sided<br>*Only common tests should be described solely by name; describe more complex techniques in the Methods section.*
☒	☐	A description of all covariates tested
☐	☒	A description of any assumptions or corrections, such as tests of normality and adjustment for multiple comparisons
☐	☒	A full description of the statistical parameters including central tendency (e.g. means) or other basic estimates (e.g. regression coefficient) AND variation (e.g. standard deviation) or associated estimates of uncertainty (e.g. confidence intervals)
☐	☒	For null hypothesis testing, the test statistic (e.g. *F*, *t*, *r*) with confidence intervals, effect sizes, degrees of freedom and *P* value noted<br>*Give P values as exact values whenever suitable.*
☒	☐	For Bayesian analysis, information on the choice of priors and Markov chain Monte Carlo settings
☒	☐	For hierarchical and complex designs, identification of the appropriate level for tests and full reporting of outcomes
☐	☒	Estimates of effect sizes (e.g. Cohen's *d*, Pearson's *r*), indicating how they were calculated

Our web collection on statistics for biologists contains articles on many of the points above.

Software and code

Policy information about availability of computer code

Data collection	When possible, behavioral tests were recorded and analyzed using AnyMaze software (AnyMaze™ 6.1 Stoelting Co.). Fluorescent images were analysed with pre-existing plugins in Fiji ImageJ as described in methods.
Data analysis	Statistical comparisons were performed using GraphPad Prism 9 software. Each dataset was tested for normality (Shapiro-Wilk test, alpha = 0.05) and outliers (Grubb's test, alpha = 0.05). Animals identified as outliers in two or more distinct behavioral measures were removed from further analysis. Two-group comparisons were performed using two-tailed unpaired Welch's t-test (normal distribution) or Mann-Whitney U-test (non-gaussian distribution). Multiple group comparisons were assessed with one- and two-way analysis of variance (ANOVA) or multiple permutations (non-equal variances) followed by Bonferroni post-hoc testing (normal distribution) or Kruskal-Wallis test with Dunn's post-hoc test (non-gaussian distribution). Principal component analysis (PCA) was performed using the R software, package FactoMineR, and missing values imputed with missMDA.

For manuscripts utilizing custom algorithms or software that are central to the research but not yet described in published literature, software must be made available to editors and reviewers. We strongly encourage code deposition in a community repository (e.g. GitHub). See the Nature Portfolio guidelines for submitting code & software for further information.

Data

Policy information about availability of data

All manuscripts must include a data availability statement. This statement should provide the following information, where applicable:

- Accession codes, unique identifiers, or web links for publicly available datasets
- A description of any restrictions on data availability
- For clinical datasets or third party data, please ensure that the statement adheres to our policy

All data supporting the findings of this study are available within the paper and Supplementary Information files

Research involving human participants, their data, or biological material

Policy information about studies with human participants or human data. See also policy information about sex, gender (identity/presentation), and sexual orientation and race, ethnicity and racism.

Reporting on sex and gender	Blood samples from human individuals with major depressive disorder and healthy controls were grouped by self-reported biological sex for analysis (Figure 7). No information was available for gender.
Reporting on race, ethnicity, or other socially relevant groupings	N/A
Population characteristics	Subjects with known history of drug abuse were excluded. Demographic characteristics associated with each sample are listed in Supp.Table 3 (sex was self-reported). Depressive behaviours were assessed by the Patient Health Questionnaire (PHQ-9), which scores each of the nine Diagnostic and Statistical Manual of Mental Disorders (DSM) IV criteria (Ref. 104).
Recruitment	Samples from volunteers with major depressive disorder were collected at the emergency room of the Institut universitaire en santé mentale de Montréal of CIUSSS de l'Est-de-Montreal and samples from healthy volunteers at the CR-IUSMM. All donors provided informed consent and signed a 7-page document detailing the goals of the Signature Bank, participants involvement (questionnaires and tissue sampling), advantages vs risks, compensation, confidentiality measures, rights as participant and contact information.
Ethics oversight	All human blood samples were provided by the Signature Bank from the Centre de recherche de l'Institut universitaire en santé mentale de Montréal (CR-IUSMM) under approval of the institution's Ethics Committee. All experiments performed using these samples were performed under the approval of Université Laval and CERVO Brain Research Center Ethics Committee 'Neurosciences et santé mentale' (Project #2019-1540).

Note that full information on the approval of the study protocol must also be provided in the manuscript.

Field-specific reporting

Please select the one below that is the best fit for your research. If you are not sure, read the appropriate sections before making your selection.

☒ Life sciences ☐ Behavioural & social sciences ☐ Ecological, evolutionary & environmental sciences

For a reference copy of the document with all sections, see nature.com/documents/nr-reporting-summary-flat.pdf

Life sciences study design

All studies must disclose on these points even when the disclosure is negative.

Sample size	Sample size for chronic social defeat stress (CSDS) mouse cohorts was calculated based on previous studies of CSDS and depression-like behaviors (Ref. 30). For viral manipulations mouse cohorts were calculated based on our previous studies (Refs. 9, 43). At least 3 replicates were performed for in vitro experiments. Number of human samples was based on previous publications (Refs. 9, 38).
Data exclusions	Outliers were detected using Grubb's test with alpha = 0.05. Individual outliers were excluded from data sets for analysis. Animals identified as outliers in two or more distinct behavioral measures were removed from all subsequent analysis and tissue processing.
Replication	Behavioral and molecular findings for CSDS and CVS were replicated in at least 2 separate cohorts of animals. Similarly, in vitro experiments were successfully repeated in at least three replicate wells, from two independent experiments.
Randomization	All animals were randomly assigned to CSDS, AAV manipulation, PE and antidepressant treatment experiments using Excel spreadsheet random function. In vitro treatment was randomly assigned to cells in culture.
Blinding	Social interaction test screening and behavioral tests were performed with automated tracking systems when possible. If not, scoring was done by experimenters blinded to experimental conditions (for splash test, sucrose preference test and forced swim test). Image analysis was performed in batches when possible, and where manual analyses were performed, experimenters were blinded to treatment condition.

Reporting for specific materials, systems and methods

We require information from authors about some types of materials, experimental systems and methods used in many studies. Here, indicate whether each material, system or method listed is relevant to your study. If you are not sure if a list item applies to your research, read the appropriate section before selecting a response.

Materials & experimental systems

n/a	Involved in the study
☐	☒ Antibodies
☐	☒ Eukaryotic cell lines
☒	☐ Palaeontology and archaeology
☐	☒ Animals and other organisms
☒	☐ Clinical data
☒	☐ Dual use research of concern
☒	☐ Plants

Methods

n/a	Involved in the study
☒	☐ ChIP-seq
☒	☐ Flow cytometry
☒	☐ MRI-based neuroimaging

Antibodies

Antibodies used

Primary Antibodies:
Cd31 Invtrogen 14-0311-85 Rat 1:100 (IF)
Cldn5 Invitrogen 34-1600 Rabbit 1:250 (IF)
Fgf2 Biosensis 10782-612 Sheep 1:200 (IF)
p(ser9)-Gsk3β Cell Signalling 9336 Rabbit 1:1000 (WB)
Gsk3β Cell Signalling 9315 Rabbit 1:1000 (WB)
p-β-Catenin Cell Signalling 9562 Rabbit 1:1000 (WB)
β-Catenin Cell Signalling 9561 Rabbit 1:1000 (IF,WB)
Cldn5 Invitrogen 34-1600 Rabbit 1:1000 (WB)

Secondary Antibodies:
Cy2 Anti-Rat Jackson ImmunoResearch 712-175-153 Donkey 1:400 (IF)
Cy3 Anti-Rabbit Jackson ImmunoResearch 711-225-152 Donkey 1:400 (IF)
Cy5 Anti-Sheep Jackson ImmunoResearch 713-175-147 Donkey 1:400 (IF)
Anti-Rabbit IgG, HRP Cell Signalling 7074 Goat 1:5000 (WB)

Validation

Validation from manufacturers website, our previous work and other relevant publications as cited throughout the text.

Eukaryotic cell lines

Policy information about cell lines and Sex and Gender in Research

Cell line source(s)

HBEC-5i (ATCC CRL-3245) - Human brain microvascular endothelial cells (sex not specified).
bEnd.3 (ATCC CRL-2299) - Mouse brain endothelial cells (sex not specified)

Authentication

Cell lines were authenticated by manufacturer as described on their websites below. Expression of relevant endothelial markers (CD31, Cldn5) was performed following protocol outlined in methods.

HBEC-5i - https://www.atcc.org/products/crl-3245
bEnd.3 - https://www.atcc.org/products/crl-2299

Mycoplasma contamination

All cell lines were confirmed negative for mycoplasma testing by manufacturer according to certificates of analysis.

Commonly misidentified lines
(See ICLAC register)

n/a

Animals and other research organisms

Policy information about studies involving animals; ARRIVE guidelines recommended for reporting animal research, and Sex and Gender in Research

Laboratory animals

Male and female C57BL/6 mice aged 8 weeks of age at arrival (Charles River 944 Laboratories, Québec, Canada) were used for all experiments. Retired male CD-1 breeders were 945 used as resident aggressors (AGG) for social defeat and social interaction tests.

Wild animals

No wild animals were used in this study.

Reporting on sex

Male and female mice were used in separate experiments. Sex is reported along with N for applicable experiments.

Field-collected samples	The study did not involve samples collected from the field.
Ethics oversight	All experimental procedures were approved by the animal care and use committee of Université Laval (2022-1061-1) and met the guidelines set out by the Canadian Council on Animal Care.

Note that full information on the approval of the study protocol must also be provided in the manuscript.

Plants

Seed stocks	n/a
Novel plant genotypes	n/a
Authentication	n/a
